# Supplementary material for: Volcanic crisis reveals coupled magma system at Santorini and Kolumbo
Source: Nature. 2025 Sep 24;645(8082):939–45. doi: 10.1038/s41586-025-09525-7 (PMC12460173; doi:10.1038/s41586-025-09525-7)
Supplement: Supplementary file 1 — Supplementary Figs. 1–6. [file 41586_2025_9525_MOESM1_ESM.pdf]

nature portfolio

https://doi.org/10.1038/s41586-025-09525-7

Supplementary information

Volcanic crisis reveals coupled magma system at Santorini and Kolumbo

In the format provided by the authors and unedited

Supplementary Information

Volcanic crisis reveals coupled magma system at Santorini and Kolumbo

Marius Isken[1+*], Jens Karstens[2+*], Paraskevi Nomikou[3], Michelle Maree Parks[4], Vincent Drouin[4], Eleonora Rivalta[1,5], Gareth J. Crutchley[2], Mahmud Haghshenas Haghighi[6], Emilie E.E. Hooft[7], Simone Cesca[1], Thomas R. Walter[1,8], Sebastian Hainzl[1,8], Joachim Saul[1], Dimitris Anastasiou[9], Kostas Raptakis[9], Nikolai M. Shapiro[10], Jannes Münchmeyer[10], Quentin Higueret[10], Jean Soubestre[10], Florent Brenguier[10], Rebeckah S. Hufstetler[7], Kaisa R. Autumn[7], Maria Tsakiri[9], Dietrich Lange[2], Heidrun Kopp[2,11], Morelia Urlaub[2,11], María Blanch Jover[2], Jonas Preine[12], Christian Hübscher[13], Mahdi Motagh[1,10], Daniel Müller[1], Torsten Dahm[1,8], Christian Berndt[2,11]

[1]GFZ Helmholtz Centre for Geosciences, Potsdam, Germany

[2]GEOMAR Helmholtz Centre for Ocean Research Kiel, Kiel, Germany

[3]Department of Geology and Geoenvironment, National and Kapodistrian University of Athens, Athens, Greece

[4]Icelandic Meteorological Office, Reykjavík, Iceland

[5]Section of Geophysics, Department of Physics and Astronomy, Alma Mater Studiorum University of Bologna, Bologna, Italy

[6]Institute of Photogrammetry and GeoInformation, Leibniz University Hannover, Hannover, Germany

[7]Department of Earth Science, University of Oregon, Eugene, OR, USA

[8]Institute of Geosciences, University of Potsdam, Potsdam, Germany

[9]School of Rural, Surveying and Geoinformatics Engineering, National Technical University of Athens, Zographos, Greece

[10]Université Grenoble Alpes, Université Savoie Mont Blanc, CNRS, IRD, Université Gustave Eiffel, ISTerre, Grenoble, France

[11]Institute of Geosciences, Kiel University, Kiel, Germany

[12]Department of Geology and Geophysics, Woods Hole Oceanographic Institution, Woods Hole, USA

[13]Institute of Geophysics, University of Hamburg, Hamburg, Germany

*Corresponding author(s). Email(s): marius.isken@gfz.de; jkarstens@geomar.de
[+]These authors contributed equally to this work.

Table of Contents

Supplementary Fig. 1: Spatiotemporal migration of seismicity as maps and cross-sections.

Supplementary Fig. 2: Time-dependent diking model intervals between 24 January to 24 February 2025.

Supplementary Fig. 3: Co-diking geodetic modelling uncertainties and posterior probability density distributions.

Supplementary Fig. 4: Geodetic model parameters for Santorini inflation event.

Supplementary Fig. 5: Seismic velocity model used for detection and localisation of seismicity.

Supplementary Fig. 6: Seismic stations used for moment tensor inversion.

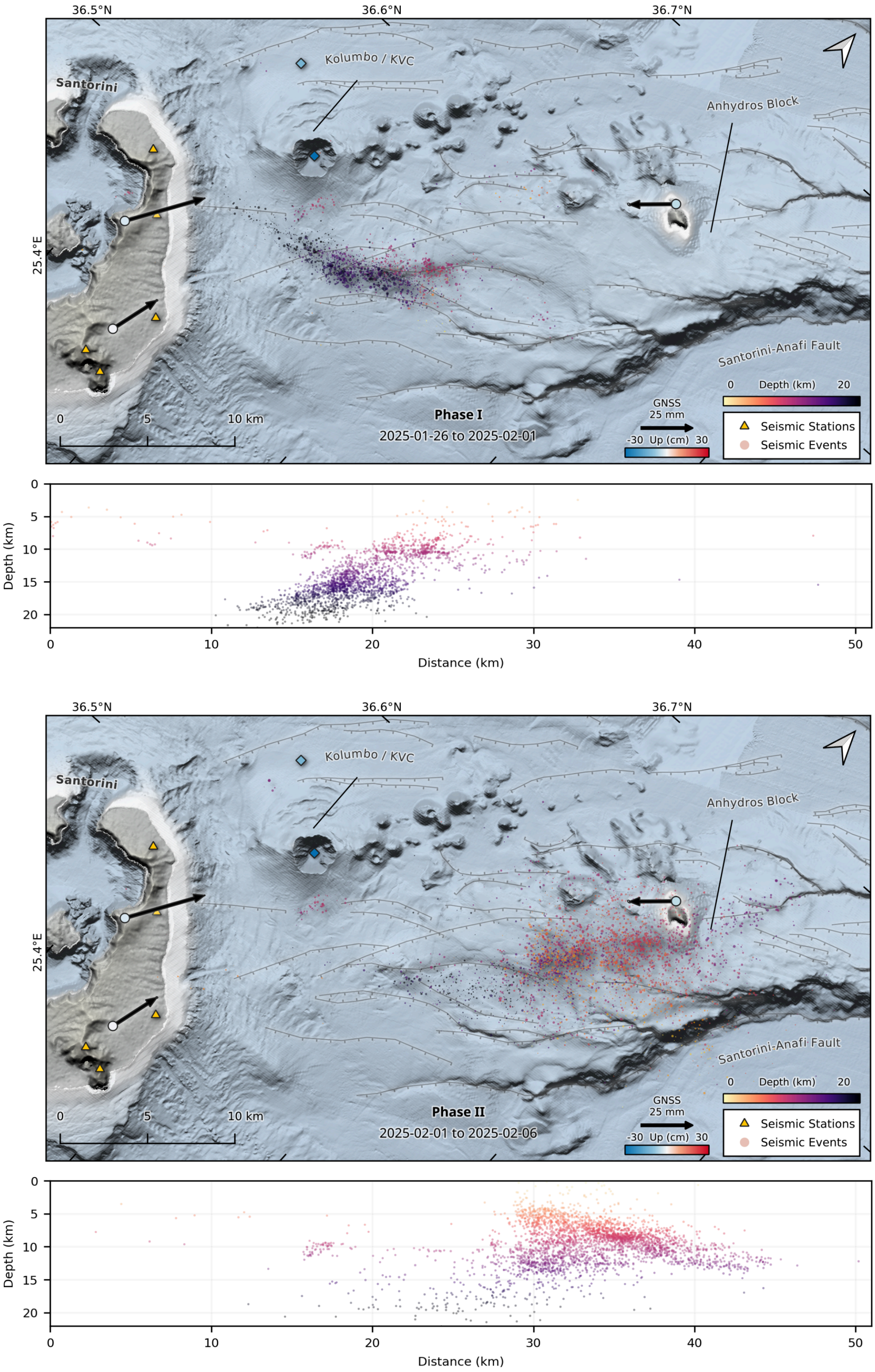
36.5°N
36.6°N
36.7°N
25.4°E
Santorini
Kolumbo / KVC
Anhydros Block
Santorini-Anafi Fault
0 Depth (km) 20
GNSS
25 mm
-30 Up (cm) 30
Seismic Stations
Seismic Events
0
5
10 km
Phase I
2025-01-26 to 2025-02-01
Depth (km)
Distance (km)
Phase II
2025-02-01 to 2025-02-06

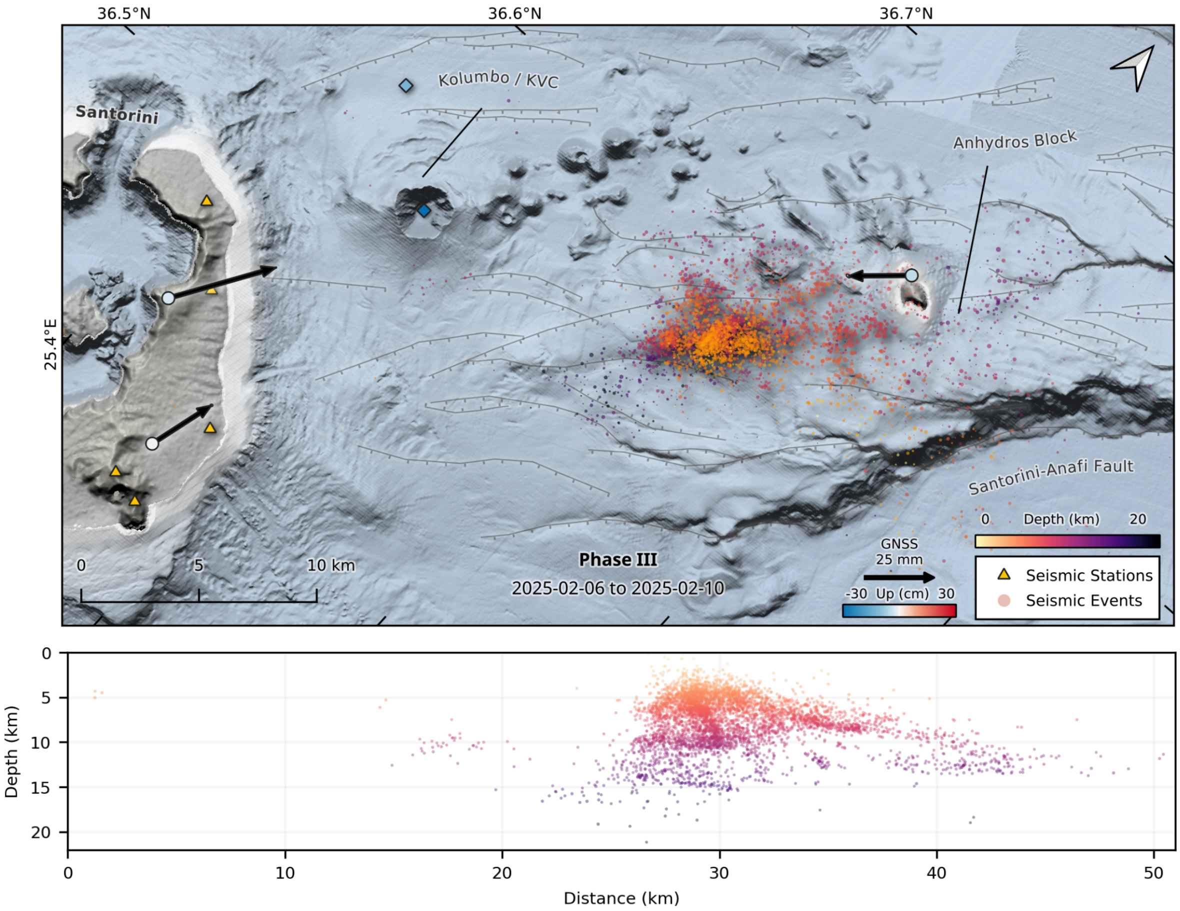
36.5°N
36.6°N
36.7°N
25.4°E
Santorini
Kolumbo / KVC
Anhydros Block
Santorini-Anafi Fault
0
5
10 km
Phase III
2025-02-06 to 2025-02-10
GNSS
25 mm
-30 Up (cm) 30
0 Depth (km) 20
Seismic Stations
Seismic Events
Depth (km)
Distance (km)

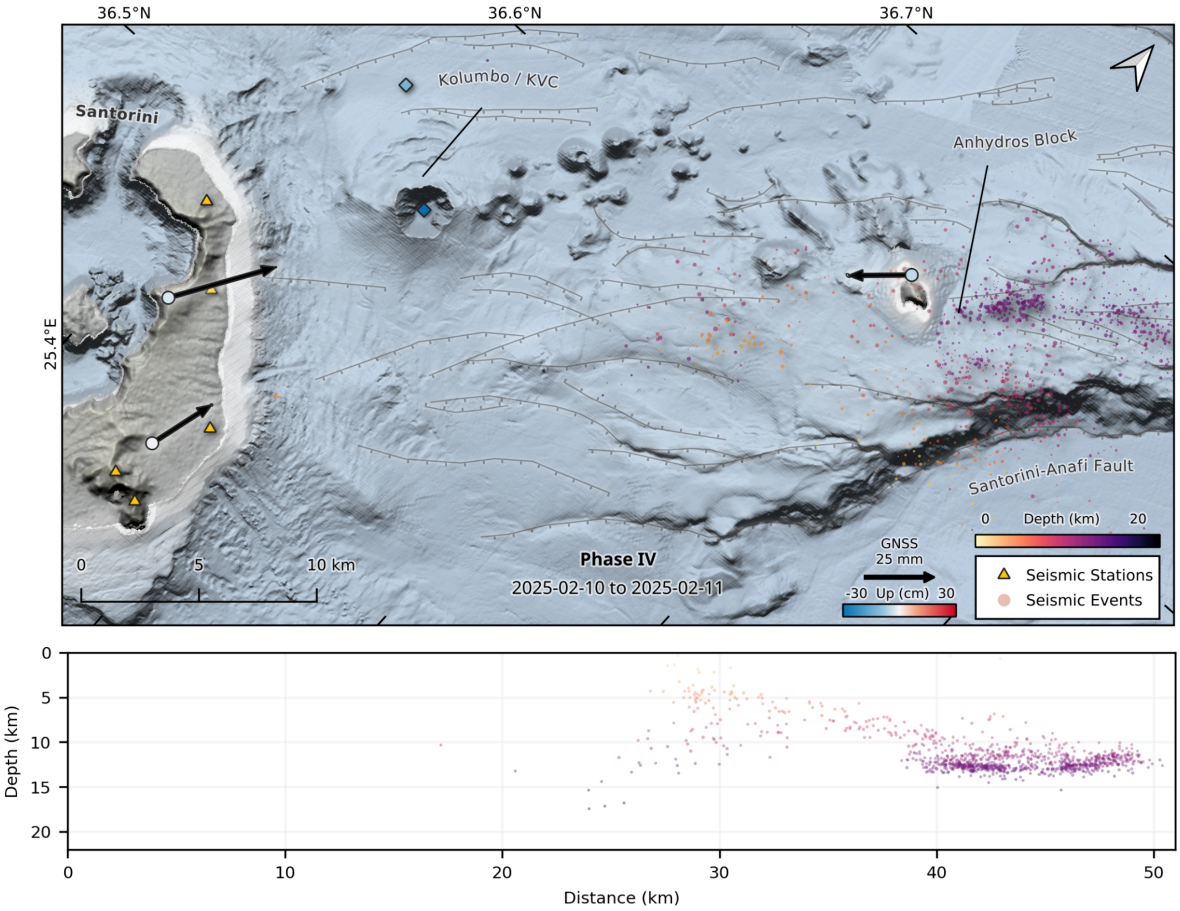
36.5°N
36.6°N
36.7°N
25.4°E
Santorini
Kolumbo / KVC
Anhydros Block
Santorini-Anafi Fault
0
5
10 km
Phase IV
2025-02-10 to 2025-02-11
GNSS
25 mm
-30 Up (cm) 30
0 Depth (km) 20
Seismic Stations
Seismic Events
Depth (km)
Distance (km)

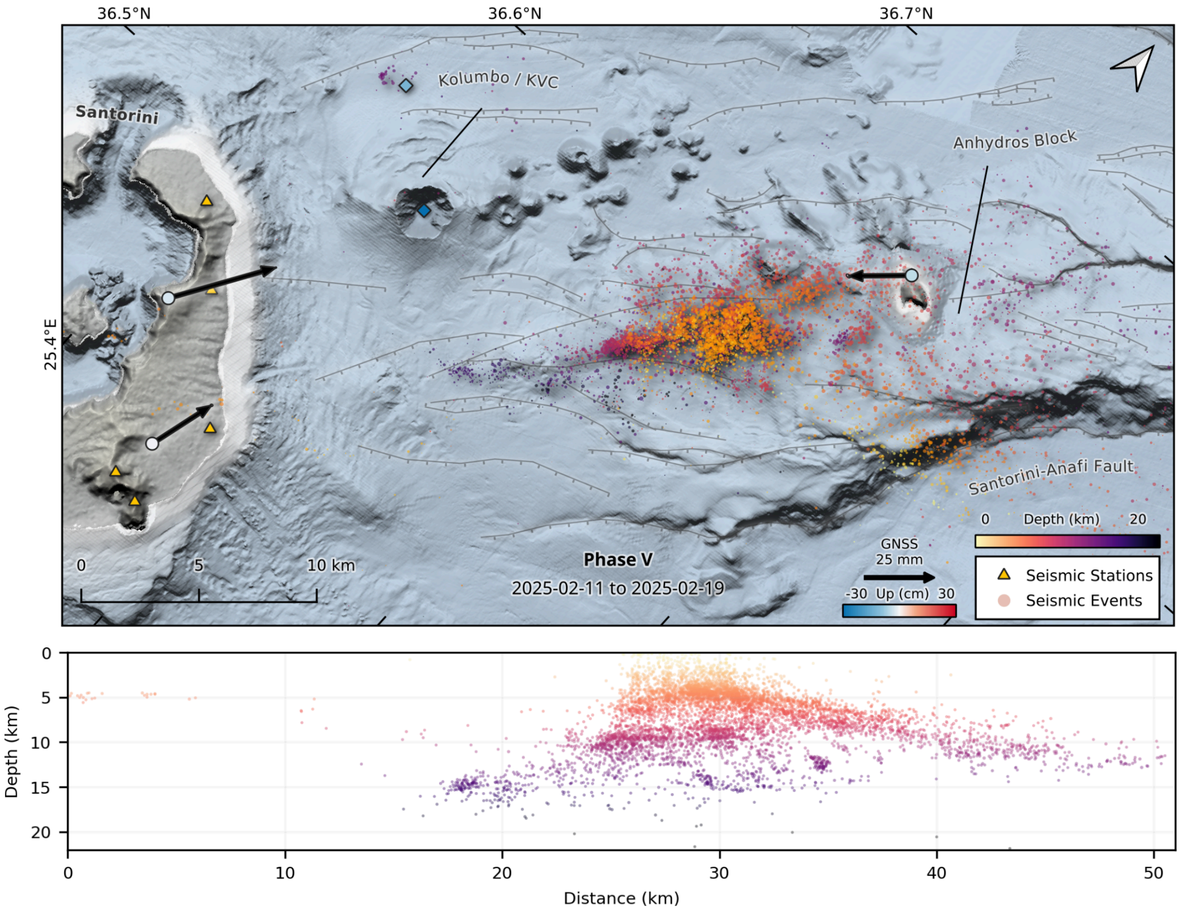

Supplementary Fig. 1 | Spatiotemporal migration of seismicity. *Maps and cross-sections illustrating the spatial migration of seismicity within phases I to V, between 24 January and 23 February 2025.*

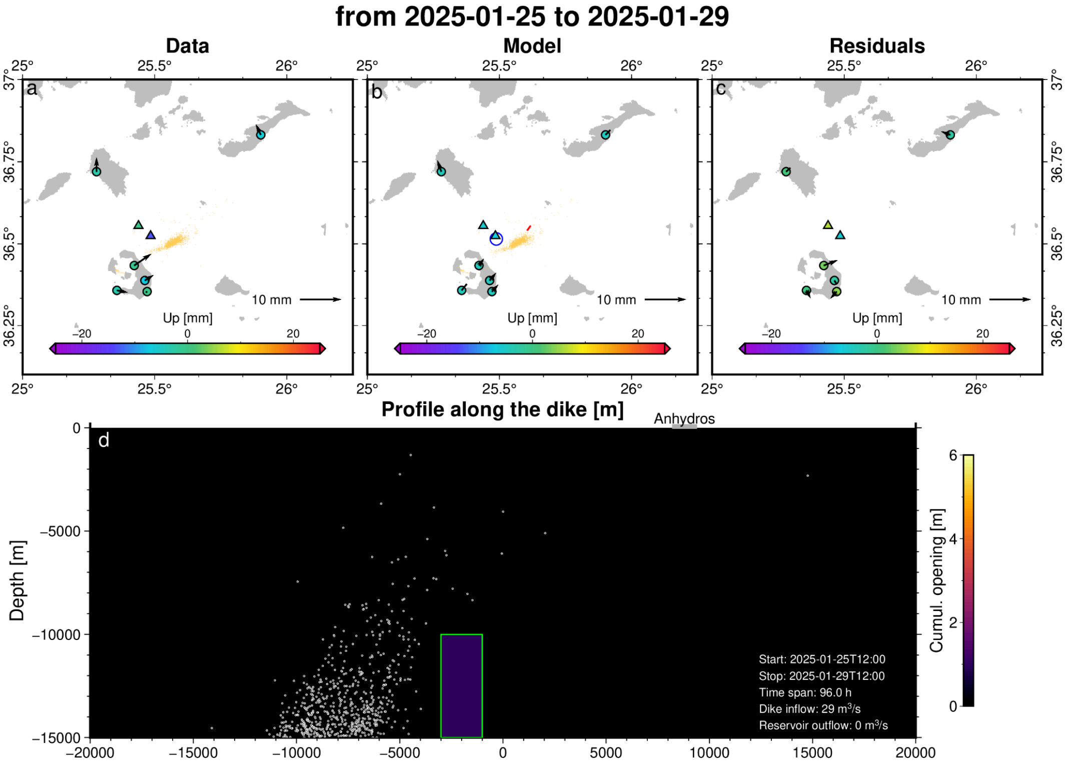
from 2025-01-25 to 2025-01-29
Data
Model
Residuals
a
b
c
10 mm
Up [mm]
−20
0
20
25°
25.5°
26°
36.25°
36.5°
36.75°
37°
Profile along the dike [m]
Anhydros
d
Depth [m]
0
−5000
−10000
−15000
−20000
−15000
−10000
−5000
0
5000
10000
15000
20000
Cumul. opening [m]
6
4
2
0
Start: 2025-01-25T12:00
Stop: 2025-01-29T12:00
Time span: 96.0 h
Dike inflow: 29 m³/s
Reservoir outflow: 0 m³/s

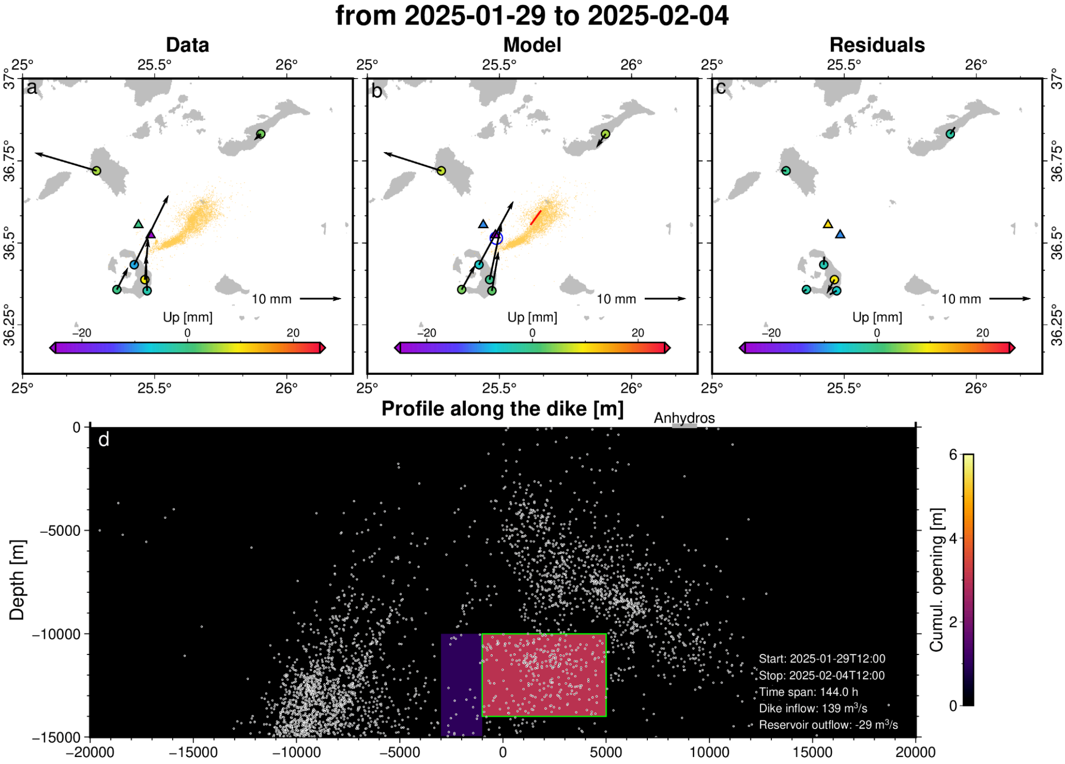
from 2025-01-29 to 2025-02-04
Data
Model
Residuals
a
b
c
10 mm
Up [mm]
−20
0
20
25°
25.5°
26°
36.25°
36.5°
36.75°
37°
Profile along the dike [m]
Anhydros
d
Depth [m]
0
−5000
−10000
−15000
−20000
−15000
−10000
−5000
0
5000
10000
15000
20000
Cumul. opening [m]
6
4
2
0
Start: 2025-01-29T12:00
Stop: 2025-02-04T12:00
Time span: 144.0 h
Dike inflow: 139 m³/s
Reservoir outflow: -29 m³/s

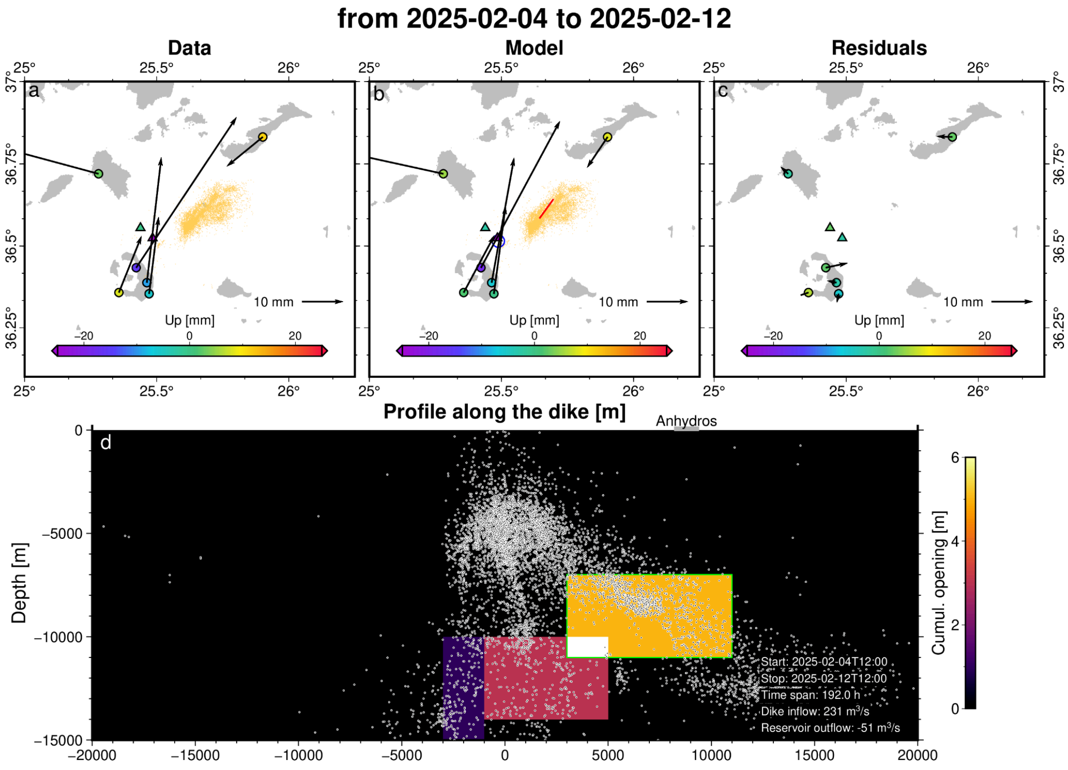
from 2025-02-04 to 2025-02-12
Data
Model
Residuals
a
b
c
10 mm
Up [mm]
Profile along the dike [m]
Anhydros
d
Depth [m]
Cumul. opening [m]
Start: 2025-02-04T12:00
Stop: 2025-02-12T12:00
Time span: 192.0 h
Dike inflow: 231 m³/s
Reservoir outflow: -51 m³/s

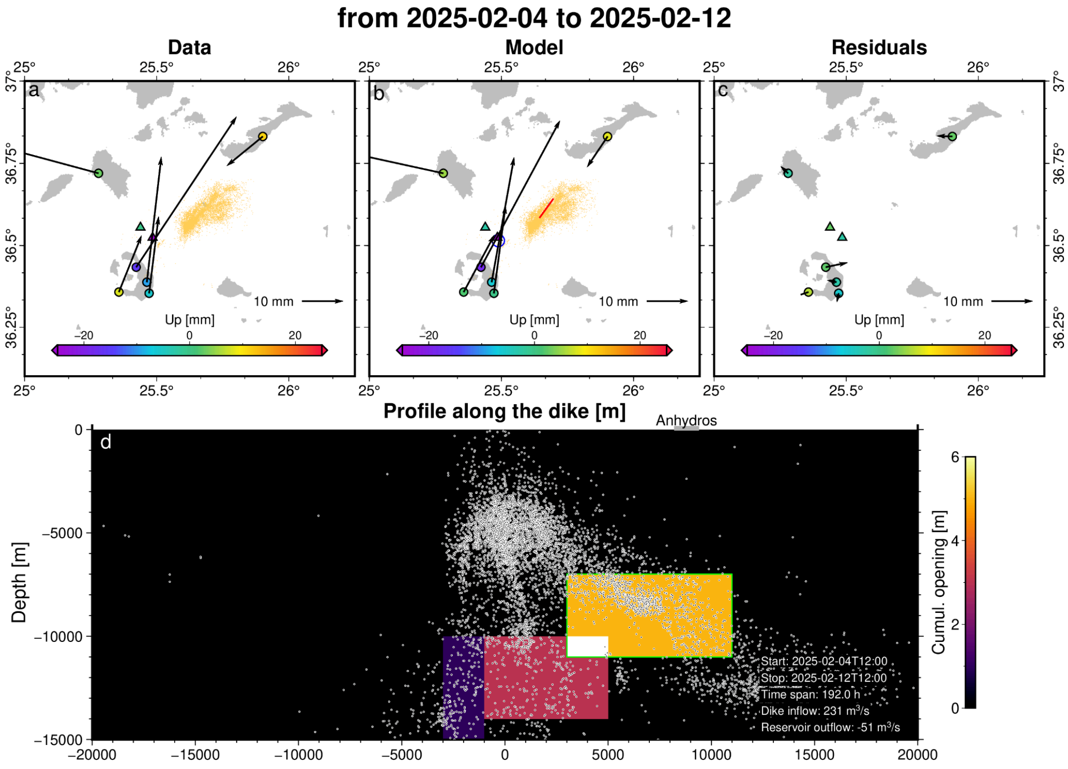
from 2025-02-04 to 2025-02-12
Data
Model
Residuals
a
b
c
10 mm
Up [mm]
Profile along the dike [m]
Anhydros
d
Depth [m]
Cumul. opening [m]
Start: 2025-02-04T12:00
Stop: 2025-02-12T12:00
Time span: 192.0 h
Dike inflow: 231 m³/s
Reservoir outflow: -51 m³/s

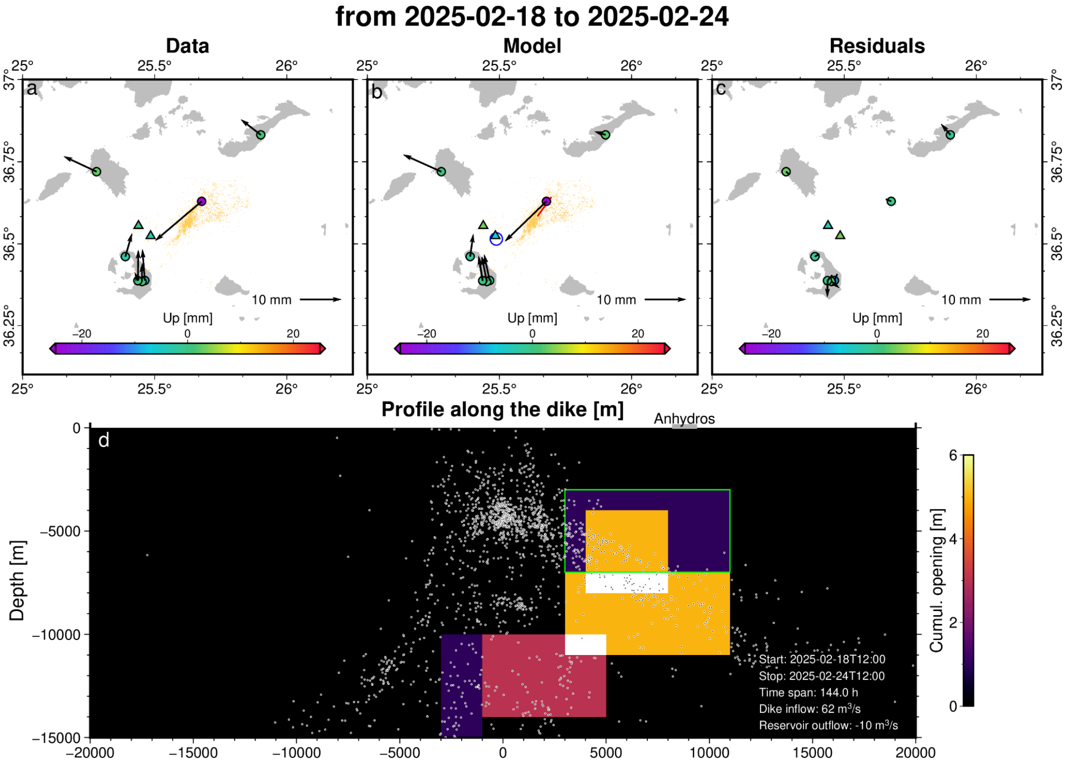

Supplementary Fig. 2: Time-dependent diking model in five time windows between 24 January to 24 February 2025. *Five time windows modelling the deflation source south of Kolumbo and the inflating dike. For each time step, the depth and horizontal location of the Okada dislocation model were inverted along a fixed profile inverted from the GNSS data (top panels).*

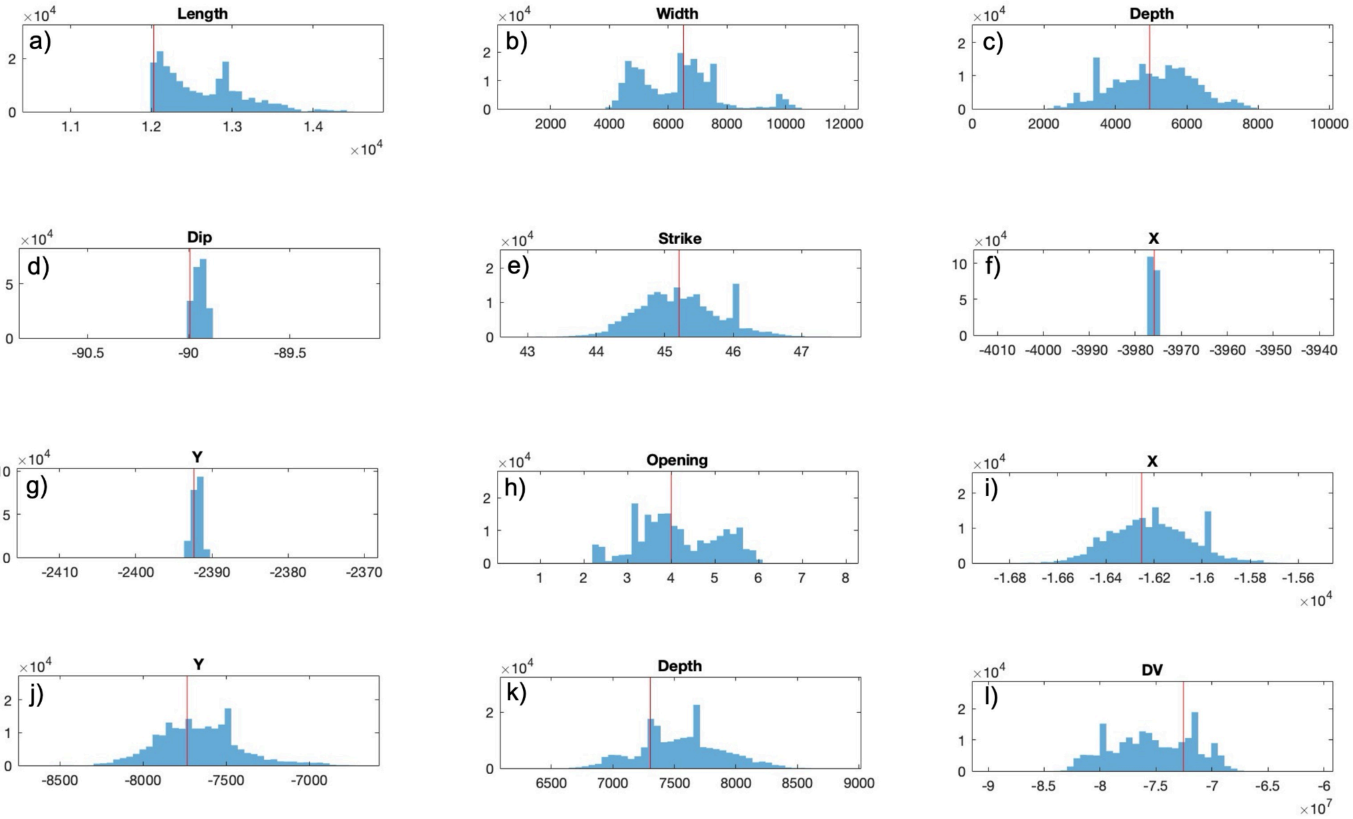

Supplementary Fig. 3 | Co-diking geodetic modelling posterior probability density distributions. *Geodetic model parameters for Kolumbo deflation and diking event spanning 18 January to 23 February 2025 using a point source of pressure (Mogi, 1958) and Okada dislocation (Okada, 1992).* ***a-h****, Probability density functions for the dike (Okada dislocation) parameters.* ***a****, length (m),* ***b****, width (m),* ***c****, top depth (m),* ***d****, dip (°),* ***e****, strike (°),* ***f****, center point x-location (m),* ***g****, center point y-location (m),* ***h****, opening (m). Dip and central X, Y coordinates of the dike were fixed. Lower bound on length was set to 12 km.* ***i-l****, Probability density functions for the deflating source (Mogi) parameters.* ***i****, x-location (m),* ***j****, y-location (m),* ***k****, depth of source (m),* ***l****, volume change (m^3). The georeference point for the model is 25.669°E, 36.584°N.*

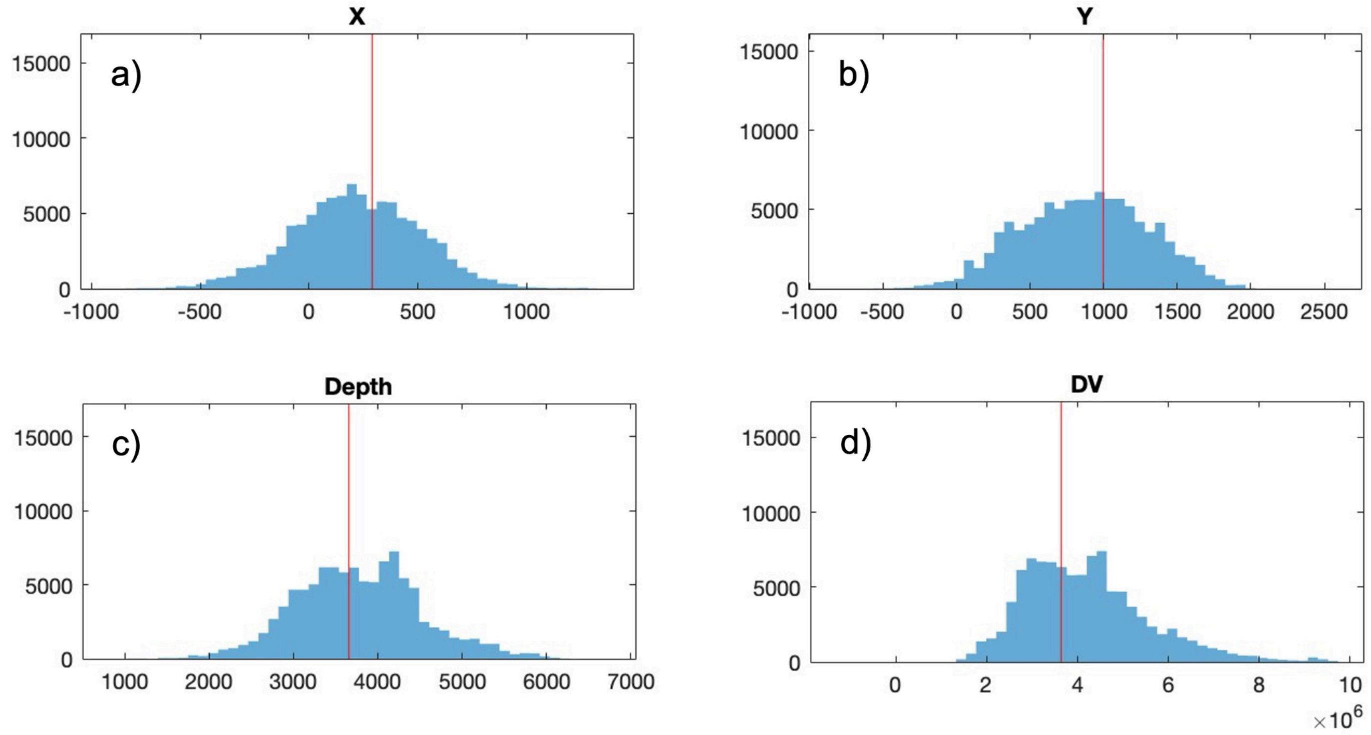

Supplementary Fig. 4 | Santorini inflation pre-diking geodetic modelling posterior probability density distributions. *Spanning 10 July 2024 to 18 January 2025 using a point source of pressure (Mogi, 1958). (a-d) Probability density functions for the source parameters.* ***a****, x-location (m),* ***b****, y-location (m),* ***c****, depth of source (m),* ***d****, volume change (m^3). The georeference point for the model is 25.387°E, 36.424°N.*

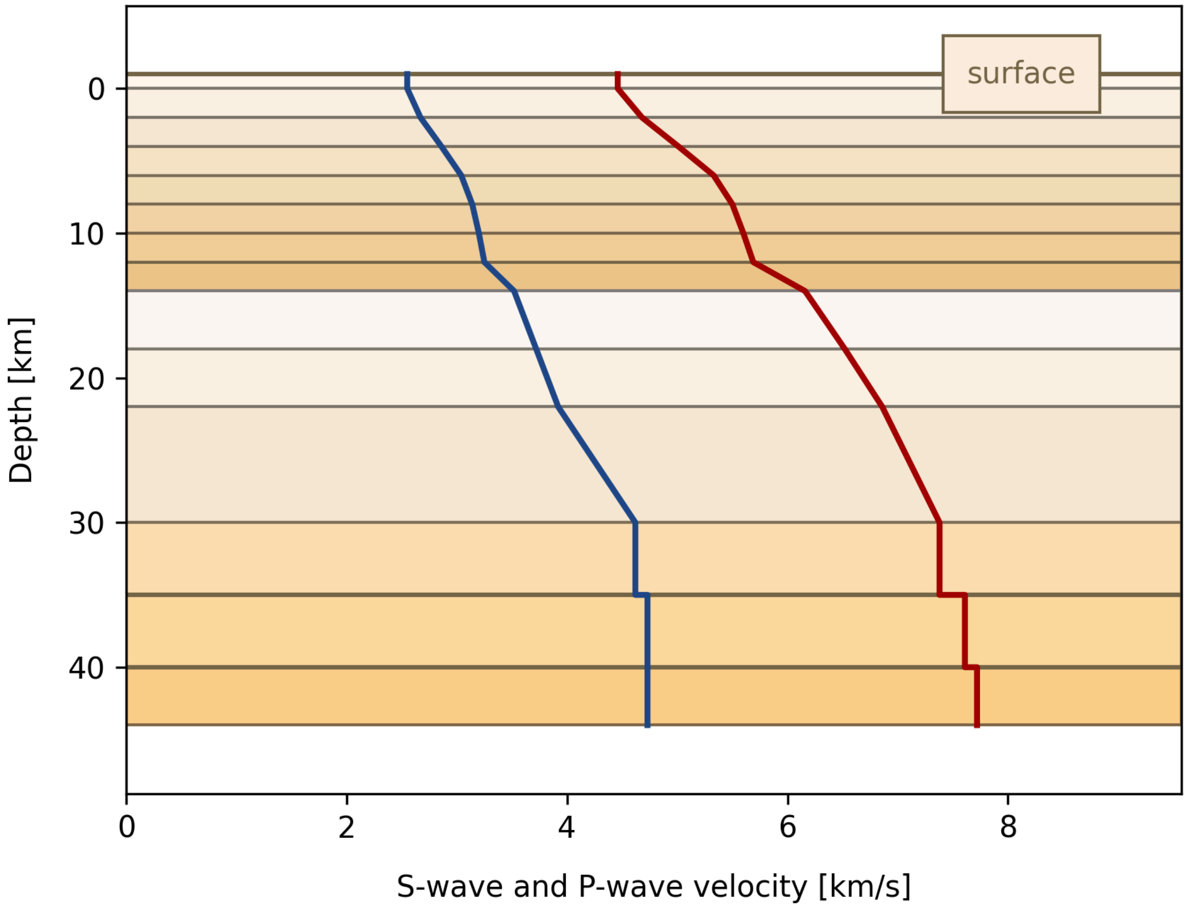

Supplementary Fig. 5 | Seismic velocity model used for detection and localisation of seismicity. *Local subsurface seismic velocity P- (red) and S-wave (blue) model used for earthquake detection and localisation. From Papadimitriou et al (2015).*

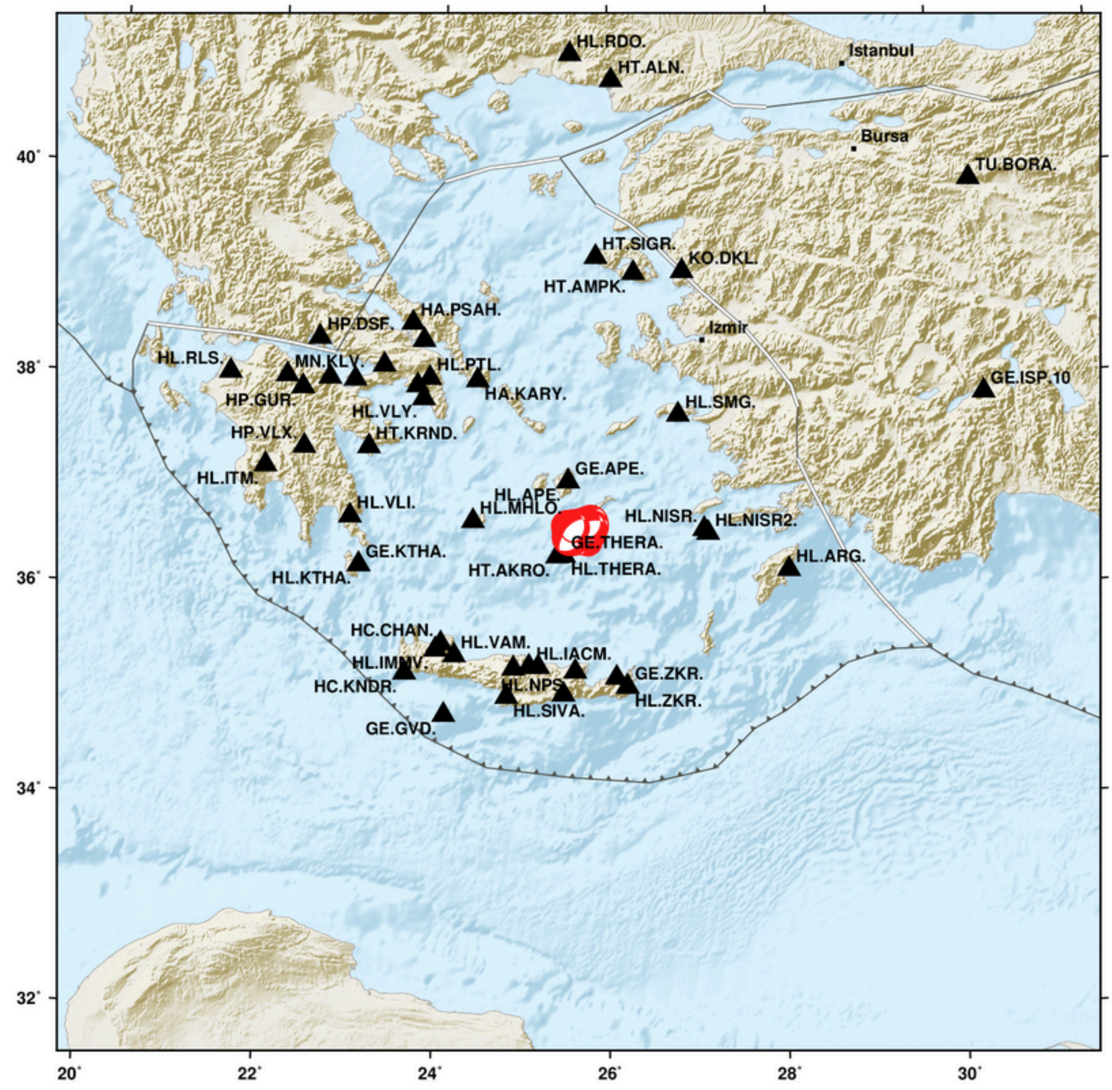

Supplementary Fig. 6 | Seismic stations used for moment tensor inversion. *The probabilistic moment tensors were inverted from a set of regional seismic stations with a maximum distance of 700 km to Santorini using the AK135 velocity model.*

References

Papadimitriou, P. et al. The Santorini Volcanic Complex: A detailed multi-parameter seismological approach with emphasis on the 2011–2012 unrest period. Journal of Geodynamics 85, 32–57 (2015).
